# BiofOmics: A Web Platform for the Systematic and Standardized Collection of High-Throughput Biofilm Data

**DOI:** 10.1371/journal.pone.0039960

**Published:** 2012-06-29

**Authors:** Anália Lourenço, Andreia Ferreira, Nuno Veiga, Idalina Machado, Maria Olivia Pereira, Nuno F. Azevedo

**Affiliations:** 1 IBB - Institute for Biotechnology and Bioengineering, Centre of Biological Engineering, University of Minho, Braga, Portugal; 2 LEPAE – Laboratory for Process, Environmental and Energy Engineering, Department of Chemical Engineering, Faculty of Engineering, University of Porto, Porto, Portugal; University of Malaya, Malaysia

## Abstract

**Background:**

Consortia of microorganisms, commonly known as biofilms, are attracting much attention from the scientific community due to their impact in human activity. As biofilm research grows to be a data-intensive discipline, the need for suitable bioinformatics approaches becomes compelling to manage and validate individual experiments, and also execute inter-laboratory large-scale comparisons. However, biofilm data is widespread across *ad hoc*, non-standardized individual files and, thus, data interchange among researchers, or any attempt of cross-laboratory experimentation or analysis, is hardly possible or even attempted.

**Methodology/Principal Findings:**

This paper presents BiofOmics, the first publicly accessible Web platform specialized in the management and analysis of data derived from biofilm high-throughput studies. The aim is to promote data interchange across laboratories, implementing collaborative experiments, and enable the development of bioinformatics tools in support of the processing and analysis of the increasing volumes of experimental biofilm data that are being generated. BiofOmics’ data deposition facility enforces data structuring and standardization, supported by controlled vocabulary. Researchers are responsible for the description of the experiments, their results and conclusions. BiofOmics’ curators interact with submitters only to enforce data structuring and the use of controlled vocabulary. Then, BiofOmics’ search facility makes publicly available the profile and data associated with a submitted study so that any researcher can profit from these standardization efforts to compare similar studies, generate new hypotheses to be tested or even extend the conditions experimented in the study.

**Significance:**

BiofOmics’ novelty lies in its support to standardized data deposition, the availability of computerizable data files and the free-of-charge dissemination of biofilm studies across the community. Hopefully, this will open promising research possibilities, namely the comparison of results between different laboratories, the reproducibility of methods within and between laboratories, and the development of guidelines and standardized protocols for biofilm formation operating procedures and analytical methods.

## Introduction

Since the 1970’s, microorganisms are known to live predominantly adhered and/or together in consortia known as biofilms [1]. A biofilm is a three-dimensional structure composed not only of microbial cells, but also of a self-produced protective matrix containing polysaccharides, proteins and other types of molecules [2]. Indeed, the formation of biofilms is a prominent example of microbial strategies to survive and adapt to (antagonistic) environmental changes.

**Figure 1:**
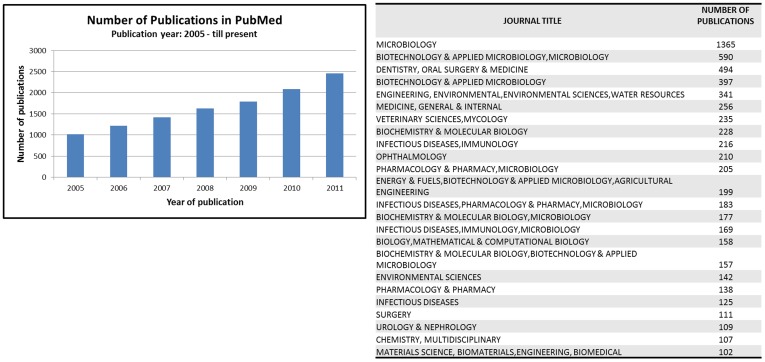
General statistics concerning publications on biofilms The plot shows the publication rate from 2000 to 2011, and the table indicates the main scope of the top 25 journals that are publishing works about biofilms.

The significance and urgency in understanding these biological structures is directly dependent on the impact that biofilms have over human activities. The increasing virulence, persistence and resistance of biofilm cells to antimicrobial agents, namely disinfectants and antibiotics, raise serious concerns in clinical, industrial and environmental settings. Biofilm-growing microorganisms are responsible for medical conditions as important as cystic fibrosis pneumonia, dental caries, and urinary catheter cystitis [3], affect hygiene and food safety in the food industry [4], and are a cause for clogging and contamination in drinking water systems [5], [6]. On the other hand, biofilms play a crucial role in the ecological balance of the Earth and can be “engineered” to carry out beneficial tasks in several biotechnological and bioengineering processes, such as wastewater treatment, bioremediation and production of biocompounds in reactors [7], [8]. Just as an illustrative example, by querying PubMed records for publications related to the term ‘biofilm’ in the last years, it can be observed that the publication rate is steadily increasing and the field attracts the attention of various communities in Microbiology, Medicine, Engineering and Environmental Sciences, among others (Figure 1).

As other domains, the emergence of high-throughput technologies has boosted biofilm research significantly. Specifically, the development of high-throughput biofilm formation platforms, e.g. microtitre-based devices [9], and advanced methods of analysis, relying on automated spectrophotometry and microscopy systems, have enabled the simultaneous testing of a wide number of variables and conditions and the production of large (statistically significant) volumes of diverse, complementary data. Moreover, the so-called “omics” platforms are promoting the comprehension of the transcriptome, proteome and metabolome of biofilms [10]–[13].

Biofilm studies are now data-intensive and researchers manifest a growing need for computational aid to both manage and analyze the data. Yet, unlike other domains, biofilm data is not accommodated in common deposition sites and there are no data interchange protocols. Apart from a small subset of data (mostly general statistics) that is presented in scientific publications, biofilm data remains in the private data files of researchers. These data files hardly follow any standards in terms of nomenclature or data structure, and have little documentation about contents. Often, experiments involve different data processing (e.g. in some experiments logarithms are applied to achieve data smoothing while in others some calculations may have to be adjusted to account for dilutions) and use different data metrics (e.g. the concentration of antibiotics can be presented either as mg/L or mol/L and data referring to the number of cells attached can be presented as either total cells per well or per cm^2^). Indeed, most data files are hardly interpretable without the help of their creators.

**Figure 2:**
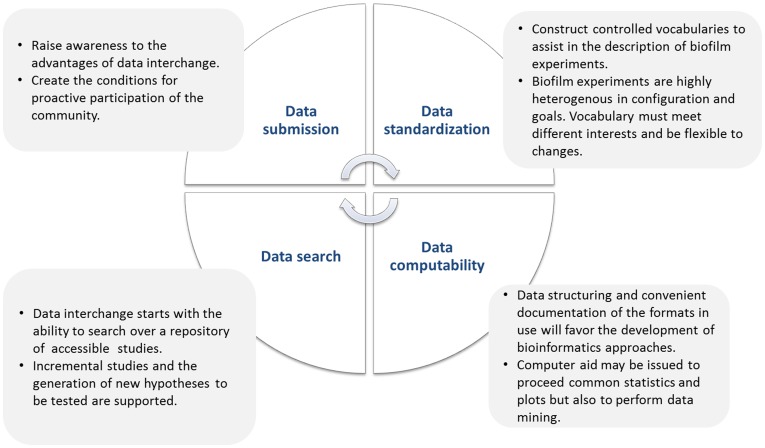
Main requirements to the development and implementation of biofilm-centered bioinformatics.

Public access to biofilm data, data standardization (both nomenclature and structure) and data integration (combining different layers of information) are requirements to the development of bioinformatics tools (Figure 2). Because data are private, tools can do little with individual studies beyond the computation of general statistics that any statistical software already does. The availability of biofilm studies opens the way to compare results across experiments and the generation of new hypotheses to complement existing data. Data documentation is required to allow for data comparison, i.e. data should follow similar processing and analysis to be comparable. Moreover, the definition of data interchange protocols will encourage research collaborations and free development of specialized tools.

The present work is the first addressing biofilm data interchange, introducing a novel and publicly available Web platform - BiofOmics - for the systematic and standardized collection of biofilm high-throughput data. This platform aims to support data accommodation, search and analysis in a general way. Despite the diversity of purposes of the biofilm studies, conditions tested and methods employed, the platform looks after the signatures of experiments, i.e. the minimum set of elements of information that characterize experiments and document the associated results. Signatures can be searched and compared, extrapolating or processing results from a number of experiments and, eventually, generating new experimental hypotheses.

In the next sections, the design and implementation of the platform will be detailed, identifying the main elements of information about a biofilm experiment and describing data collection and standardization. Since the team and their laboratories are involved in high-throughput research projects on biofilms, a variety of in-house experiments is used in platform testing and validation, exposing its utility (e.g. functionalities, usability, etc). The discrimination of current limitations, challenges and near-by directions of work aims to raise the awareness for active community participation on this type of endeavor. Further details on the project as well as unrestricted access to the platform are granted at http://biofomics.org, free of any charge.

## Methods

### Platform Architecture

BiofOmics is a Web-based framework. Its server runs on a CentOS platform (version 5.6) with Apache HTTP server (version 2.0), MySQL server (version 14.14, distribution 5.1.52) and PHP 5.1.6. Apache, MySQL and PHP technology were preferred as they are open-source software and platform-independent. Moreover, MySQL supports multi-threading and multi-user environments and thus, it is well-suited to support (increasing) real-world database usage.

Currently, the Web server and all parts of the database are hosted at the Centre of Biological Engineering of the University of Minho, Portugal. The host is dedicated to BiofOmics and no issues of quality of service (QoS) are envisioned for the near future. Nevertheless, it is anticipated the possible migration of the platform to a third-party hosting platform in order to guarantee the QoS of BiofOmics when user demands (namely, data volumes to be managed and the complexity of analyses) grow larger.

### Database Construction and Population

MySQL was used as a supported relational database management system (RDBMS). As most of the biofilm experiments performed in bench research vary and certain methods are employed routinely across the board, database modeling involved rounds of discussion with researchers to identify the minimum set of elements of information necessary to profile a biofilm study and describe the data associated. As far as possible, the data model has been made flexible to embrace new-to-appear biofilm-related data requirements as, for example, additional purposes of study.

BiofOmics supports online data submission, systematizing study description and generating standardized Excel files to upload the data. The platform is well-suited to compile data from different types of biofilm forming devices (e.g. 6-, 24-, 96-well microtitre plates with or without coupons, the Calgary device [14]–[16], etc.) and different state-of-the-art analytical methods (e. g. crystal violet (CV), 4',6-diamidino-2-phenylindole (DAPI), fluorescence *in situ* hybridization (FISH) [17]–[19]). The experiment profile includes the device(s) used to form the biofilms, the microorganisms involved, the growth media, the adhesion surfaces used to promote biofilm development, and any antimicrobial products that might have been tested; other continuous variables, such as physico-chemical settings (e.g. the temperature, pH and shear stress), are also considered. Furthermore, the submitter provides a short description of the study, its main findings and any associated publications.

Biofilm data is described quantitatively according to the method(s) of analysis that are used (e.g. the CV for total biofilm biomass or the DAPI for total biofilm cells assessment). The data files are structured hierarchically and are fully customized to encompass any arrangement of conditions tested and methods of analysis in use and thus, ensure its subsequent computerized processing [20].

### New Ontology

Often, researchers have their own mnemonics or some sort of name abbreviations and fail to document them well enough for the data files to be easily interpretable by others. Additionally, data files tend to be oblivious about data pre-processing (e.g. calculations considering the area of the wells or log reductions) and the units of measure used. As such, public data accommodation and interchange are conditioned not only by the willingness of researchers to make their data files available but also by intensive curation efforts that require close collaboration of researchers.

No controlled vocabulary specialized in the characterization of biofilm-related studies was available. BiofOmics team is accounting for this lacuna with the proactive collaboration of the advisory board and data submitters. A general ontology is being specified to embrace all major areas of information in biofilm experiments, from the description of devices and experimental procedures to the analytical methods and data pre-processing steps taken in results generation and analysis. This ontology, named Biofilm Science Ontology (BSO), has been divided into ontologies specialized in given topics (http://miabie.org/ontology). For example, there is the Experimental Procedure Ontology and the Colony Morphology Ontology [21].

As a means to establish a consensual and unambiguous language in support of biofilm-specific bioinformatics, most of the vocabulary is being compiled and normalized manually. Only microbial species and antimicrobial agents relate to existing databases - species to the NCBI Taxonomy Browser [22] and antimicrobial agents to the CAMP database [23], respectively. The compilation of terminology on parameters and conditions for biofilm formation and methods for biofilm analysis is being performed as needed.

**Figure 3:**
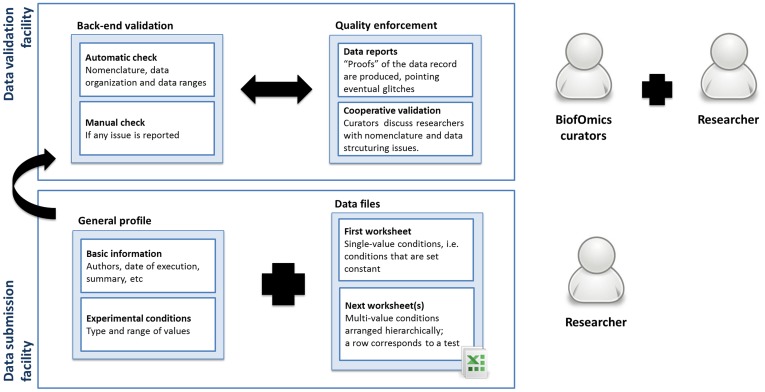
Data submission process: the data submission facility allows researchers to submit files arranged in a certain format, containing a minimum set of information. Researchers and curators work together in order to validate and ensure the quality of the submission.

## Results and Discussion

### Database Validation

Primarily, a dozen of in-house (and already published) experiments were used to validate the database model and the data submission facility. The aim was three-fold: to validate the platform by using experiments that the team could easily interpret on their own; to have a number of diverse, state-of-the-art studies in hands to figure out the best way to systematize the dissimilarities and specificities of experimental profiles, and account for high-throughput data structuring and standardization; and to load the database with real-world experiments and thus, expose to researchers the immediate advantages of submitting their data to BiofOmics, namely in terms of achieving community dissemination (and recognition) and assisting peer-review processes.

**Figure 4:**
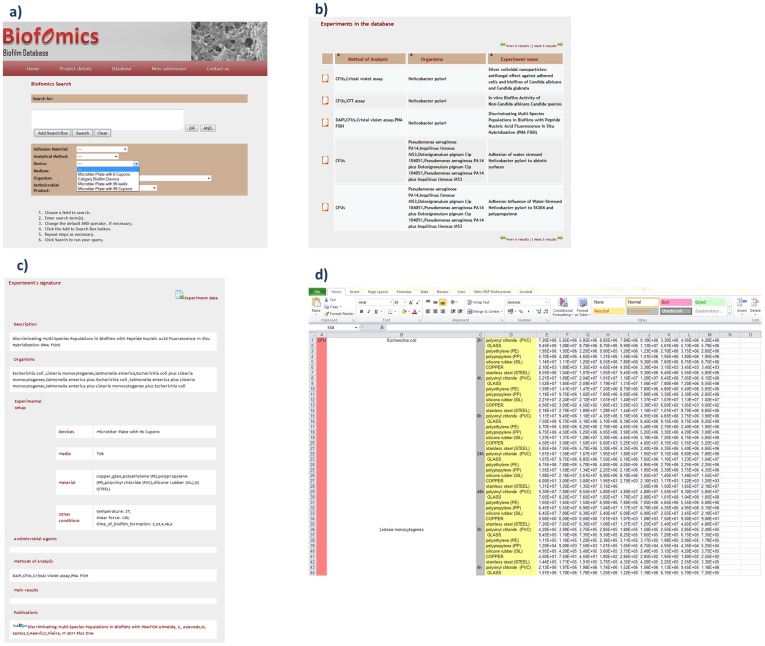
BiofOmics search facility. Researchers can issue searches over biofilm-forming devices, microbial species, growth media, adhesion material and antimicrobial products. Available search terms come from biofilms ontology and report to studies in catalogue.

The selected experiments account for representative biofilm-forming devices such as the 6-well microtitre plates with coupons, the 96-well microtitre plates and the Calgary device. More importantly, they encompass very different types of analytical methods, considered to be the most relevant in this field. Methods that are already stored in the database include crystal violet, XTT, DAPI, cultivability, FISH and Syto 9/PI. Even though some methods may seem to assess similar biofilm characteristics, for the purpose of the database, it is considered that they detect different aspects of the biofilm. For instance, despite the fact that both XTT and Syto 9/PI are considered by most authors as suitable to assess the physiological status of cells, they actually assess the respiratory activity and membrane integrity of the cells, respectively. Additionally, they are both assessed by different types of measurement devices (XTT by spectrophotometry and Syto 9/PI by microscopy), which implies that different types of dimensional units and numbers of replicates are going to be obtained in the experiments. Owing to the amount of information involved in high-throughput biofilm experiments, the number of data points that has been collected exceeds 10000.

Adding to the complexity of the devices and analytical methods, the database validation also accounted for the purpose of the experiment. As an example, experiments dealing with the resistance of biofilms to antimicrobial agents typically have data from biofilms before, during and after exposure to the agents. In this scenario, researchers will be most likely interested in comparing the values collected and for this purpose, values have to be adequately characterized in the database.

### Contents and Organization

BiofOmics team is encouraging other research groups to participate actively in database population as well as resource and tool development. External researchers are already using the data submission facility to create the customized and structured Excel data files and have interacted with BiofOmics’ curators to meet data quality issues. Additionally, they have alerted to new data requirements (e.g. when using other analytical methods or data pre-processing) and provided comments/suggestions on the vocabulary in use.

Recently, a short-term private data accommodation facility has been introduced in BiofOmics as means to assist the peer-reviewing of new manuscripts. The researcher is able to profile the experiment and detail experimental data, similarly to any public data submission. The difference is that these data can only be accessed upon authorized login (a login and password per experiment, shared with a journal’s editor to ensure anonymous access by journal reviewers).

**Figure 5:**
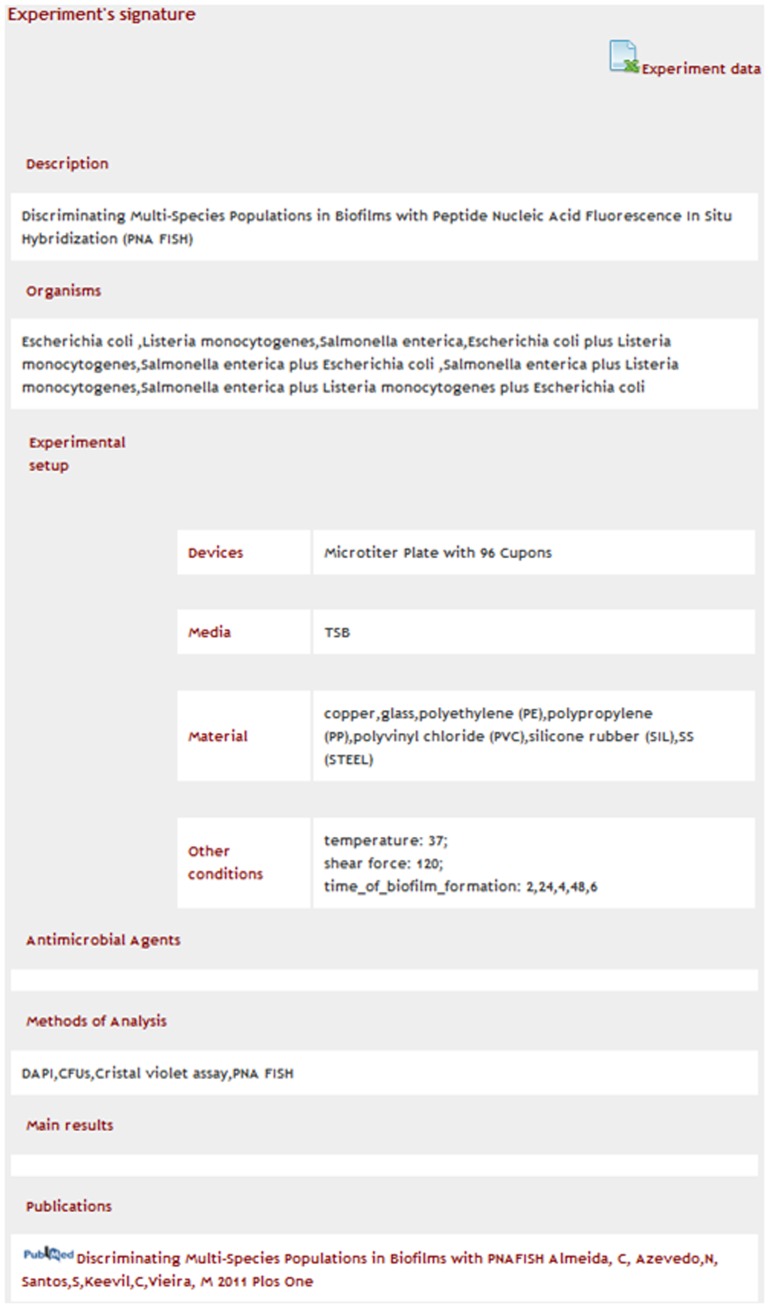
Extract of the study signature of the experiment “Discriminating multi-species populations in biofilms with peptide nucleic acid fluorescence *in situ* hybridization (PNA FISH)” (PMID: 21479268).

### Online Automatic Data Submission

BiofOmics relies on voluntary data submissions. As such, the platform is equipped with an online data submission interface that enables researchers to specify the full extent of their records –that is, to characterize the experiment in terms of aims, environmental and operational conditions and major results – and construct the structured data files to be shared with the community.

Following researcher’s specifications, BiofOmics engages a three-step protocol to profile the biofilm experiment, standardize and systematize its data, and upload the data into the database (Figure 3). Researchers do not deal with the computational technicalities, being asked only to interpret the experiment’s tests and results, and “translate” their terminology to the terminology used in BiofOmics.

Specifically, to promote the broad participation of biofilm researchers and avoid a drastic “cultural” change (most likely prone to conflicts and time consuming), BiofOmics explores the familiar format of Microsoft Excel worksheets as an intermediate, computer-amenable format between individual formats (most likely also in Excel format) and database records. Furthermore, in support of the deposition of highly specialized, atypical experiments, the platform integrates administrative processing tools.

In back-end, the curation tools check data file uploads for non-compliant nomenclature and typos, non-compliant data structuring (i.e. any alteration in the template of the worksheets), and data inconsistencies (e.g. unusual value ranges that might be indicative of the use of a different unit of measure or some data pre-processing). The researcher receives a validation report and his agreement is required to issue any corrections, either in structure or data. Similar to the publication of a manuscript, a term of responsibility and agreement is signed before data is made public.

### Data Search

As a Web-based platform, all data in BiofOmics can be accessed and retrieved directly from the Web browser. The database browse interface provides the users with a function to navigate the entire database and retrieve desired information by indicating information about the biofilm-forming microorganism, biofilm-forming device, growth medium, adhesion surface or antimicrobial product of interest (Figure 4). Queries are constructed using the terms from the ontologies and common Boolean logic.

From the list of potentially relevant biofilm studies, the researcher can access the details of any experiment, including its data and associated publications (Figure 5).

### Future Work Directions

BiofOmics novelty lays on the proposal of means to support biofilm data interchange and study comparison. For the very first time, a work focus on the documentation of biofilm studies, identifying the minimum set of elements of information necessary to produce a searchable experiment signature, and defining a systematic, structured and highly customizable approach to the creation of biofilm data files.

By promoting data interchange, BiofOmics is encouraging collaboration within and across laboratories. Researchers may issue large-scale comparisons or extend existing studies (e.g. adding new tests or extending test conditions). Laboratories may collaborate in the development of standard operating procedures or in large-scale studies of key topics. Moreover, bioinformaticians may invest in the development of a number of computer applications (e.g. model construction and simulation, antimicrobial susceptibility testing), supported by standard nomenclatures and minimum information specifications.

The construction of statistical assessment tools is one of the next logical steps in BiofOmics development. The idea is to facilitate data interpretation (possibly using a 3-D graphical interface) and enable the online comparison between experiments, avoiding the need to download all data sets and the adaptation to general analysis tools (e.g. the creation of scripts for comparative analysis of heterogeneous data).

In parallel, and similarly to what happens for microarray and proteomics experiments, where the concept of minimum information about microarrays experiments (MIAME) [24] and minimum information about proteomics experiments (MIAPE) [25] have been implemented, a set of guidelines describing the minimum information about biofilm experiments (MIABiE) is being prepared (http://miabie.org). For this purpose, Biofomics has teamed up with a group of worldwide experts in biofilms and is deploying information campaigns and debates in biofilm conferences and near journal editors. Jointly, MIABiE standards and BiofOmics tools will act not only as a facilitator for comparisons between biofilm experiments but hopefully also as a way of harmonizing methods across the biofilm area.

### Conclusions

BiofOmics (http://biofomics.org) is a publicly available framework that has been developed for the advancement of the present understanding of biofilms. It is meant to be at the core of community efforts, providing accommodation to existing data, but far more important, ensuring data standardization.

The existence of a database compiling existing biofilm data in a computer-amenable way eases research in a number of ways: the search for similar experiments, the interchange of data between researchers and laboratories, the search for “open spots” (i.e. relevant but under-reported areas), the statistical analysis of experimental robustness, ruggedness and reproducibility, and the comparative analysis of experiments (in particular, inter-laboratory collaborations). Besides the obvious value of widespread dissemination of biofilm research, researchers are also rewarded with the possibility to ameliorate the supplementary materials accompanying publications; a (major) step forward to endorse the transparency and high-quality of biofilm experimental data as well as the validity of the results and discussion being published.

To the best of our knowledge there have not been any previous attempts to standardize the documentation of biofilm studies, or centralize biofilm experimental data.
